# Evaluation of Solubility, Dissolution Rate, and Oral Bioavailability of β-Cyclodextrin and Hydroxypropyl β-Cyclodextrin as Inclusion Complexes of the Tyrosine Kinase Inhibitor, Alectinib

**DOI:** 10.3390/ph17060737

**Published:** 2024-06-05

**Authors:** Bashar J. M. Majeed, Mohammed A. Saadallah, Israa H. Al-Ani, Mohamed K. El-Tanani, Khaldun M. Al Azzam, Hassan H. Abdallah, Feras Al-Hajji

**Affiliations:** 1 Faculty of Pharmacy, Pharmacological and Diagnostic Research Center (PDRC), Al-Ahliyya Amman University, Amman 19328, Jordan; bashar.jihad@gmail.com (B.J.M.M.); eltanani@rakmhsu.ac.ae (M.K.E.-T.); 2 College of Pharmacy, RAK Medical and Health Sciences University, Ras Al-Khaimah P.O. Box 12973, United Arab Emirates; 3 Department of Chemistry, Faculty of Science, The University of Jordan, Amman 11942, Jordan; azzamkha@yahoo.com; 4 Chemistry Department, College of Education, Salahaddin University, Erbil 44002, Iraq; hwchems@yahoo.com; 5 Faculty of Pharmacy, Applied Science University, Amman 11937, Jordan; f_elhajji@asu.edu.jo

**Keywords:** alectinib, inclusion complexes, pharmacokinetics, non-small-cell lung carcinoma, bioavailability

## Abstract

This study aims to improve the solubility and dissolution rate of alectinib (ALB), a tyrosine kinase inhibitor commonly used for treating non-small-cell carcinoma (NSCLC). Given ALB’s low solubility and bioavailability, complexation with β-cyclodextrin (βCD) and hydroxy propyl β-cyclodextrin (HPβCD) was evaluated. Some of the different preparation methods used with varying ALB-to-CD ratios led to the formation of complexes that were characterized using Fourier-Transform Infrared (FTIR) techniques and Differential Scanning Calorimetry (DSC) to prove complex formation. The encapsulation efficiency was also determined. The simulations were carried out for ALB’s interactions with βCD and HPβCD. This study identified the most soluble complex (ALB–HPβCD; 1:2 ratio) and evaluated its dissolution. The bioavailability of the ALB–HPβCD complex was evaluated in Wistar rats relative to free ALB. Pharmacokinetic profiles revealed increased Cmax (240 ± 26.95 ng/mL to 474 ± 50.07 ng/mL) and AUC0-48 (5946.75 ± 265 ng.h/mL to 10520 ± 310 ng.h/mL) with no change in the elimination rate constant. In conclusion, the complexation of ALB–HPβCD manages to increase in vitro solubility, the dissolution rate, and oral bioavailability, providing a favorable approach to improving ALB administration.

## 1. Introduction

In terms of mortality, lung cancer is among the top causes of death globally [1]. However, non-small-cell lung cancer (NSCLC) is estimated to affect almost 85% of all lung cancer patients. The ranks of these subtypes belong imminently to those most commonly diagnosed symptoms and those having a negative impact on prognosis [2]. To the right of NSCLC, lung adenocarcinoma and lung squamous cell carcinoma are among other subclasses that endanger humans [3]. Both SCLC and NSCLC are caused by smoking; however, SCLC has also been found in non-smokers [4]. According to the American Cancer Society, lung cancer remains a major health challenge worldwide. As of the most recent data from 2024, lung cancer is the most commonly diagnosed cancer and the leading cause of cancer death globally. It accounts for nearly 2.5 million new cases annually, representing one in eight of all new cancer cases [5]. The incidence and mortality rates are significantly influenced by factors like smoking, which is the leading cause of lung cancer globally. Over 70% of lung cancer cases are estimated to be directly linked to tobacco use, with smoking increasing the risk of developing lung cancer by as much as 25-fold [6]. Plenty of other factors, such as ionizing radiation, patients with Hodgkin’s lymphoma, breast cancer, heavy metal toxins, pulmonary fibrosis case history, excessive alcohol consumption, and human immunodeficiency viral infections, can be the risk factors for increased lung cancer incidences [7,8,9]. Alectinib (ALB) belongs to the Anaplastic Lymphoma Kinase (ALK) inhibitor category, which belongs to the ATP–competitive drug candidates. It is a selective small molecule intended for the central nervous system (CNS) with nanomolar potency [10,11]. ALB has won considerable public favor as a second-generation ALK-tyrosine kinase inhibitor in treating ALK-positive NSCLC. ALB’s in vivo and in vitro antitumor efficacy rates were reported [12]. Significant kinase-blocking activities of ALB against ALK and resistance mutations were demonstrated in the in vitro kinase assays, where crizotinib was found to be less effective [13,14]. Due to its better CNS blood–brain barrier (BBB) crossing capacity, ALB enhanced the survival of the ALK-driven tumor cell lines implanted in the mouse models [15,16].

ALB has slight solubility in water, with a log partition coefficient of 5.2 and a pKa value of 7.05 [17]. ALB belongs to Class IV BCS (low solubility and high permeability) and is administered with food [16]. ALB has a low oral bioavailability rate of around 37% [18], and food positively affects its bioavailability, as explained by Lanser et al. [19]. The oral bioavailability of the drug molecule is based on various parameters, including drug permeability, aqueous solubility, pre-systemic metabolism, first-pass metabolism, the dissolution rate, and efflux mechanisms dependent on vulnerability. Moreover, low aqueous solubility and poor permeability are the most common causes of insignificant oral bioavailability [20,21]. Several approaches are used to enhance the bioavailability of a molecule, like the enhancement of the dissolution rate of drugs with dissolution-limited bioavailability, prodrug approach, and novel drug delivery systems that decrease drug degradation in GIT and increase the permeability of the molecules [22]. 

One of these approaches is the inclusion complexation of lipophilic drugs using cyclodextrin (CD), especially β-cyclodextrin (βCD) and hydroxypropyl-β-cyclodextrin (HPβCD), which proved its efficiency in the enhancement of solubility and oral bioavailability of slightly soluble drugs [23,24]. Ketoconazole [25], nifedipine [26], and miloxicam [27] are only a few of the many examples of lipophilic drugs whose solubility, dissolution rate, and oral bioavailability were proved to be enhanced by complexation with CD. 

The current study aims to prepare the ALB–cyclodextrin complex (βCD and HP βCD) and evaluate the prepared complex in terms of its solubility and encapsulation efficiency, along with a characterization and dissolution study. Then, an in vivo evaluation of the oral bioavailability of the complex compared to free ALB was conducted on animal model.

## 2. Results 

### 2.1. Method of Analysis

**Figure 1:**
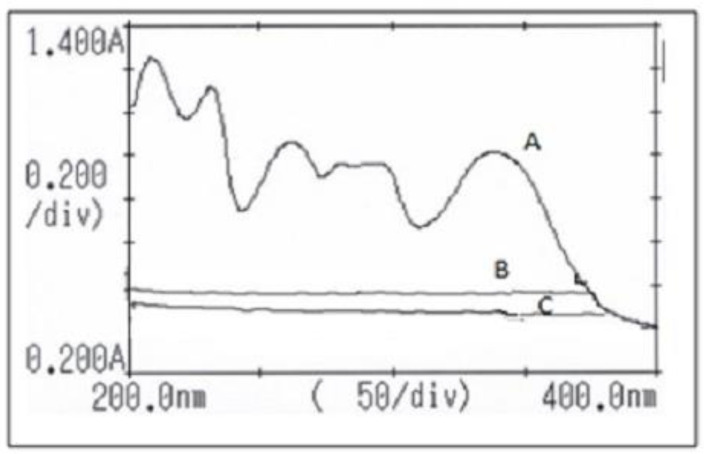
A UV scan of ALB (A) showing λmax at highest absorption, βCD (B), and HPβCD (C).

The UV scanning of ALB in methanol showed its λmax as 340 nm, and the scanning of both βCD and HPβCD showed no peaks at the same λmax of ALB; this indicated the selectivity of the method. Figure 1 shows the UV scan of ALB, βCD, and HPβCD. The linearity of this method was proven to be in the range of 15–90 µg/mL, with its R^2^ equal to 0.9939. The back calculations showed that the %RSD ranged between 0.25 and 2.6%, which complies with the specifications of the ICH guidelines.

For precision and accuracy, the RSD of day 1 was 1.2 and 0.88 for day 2 for the six measured samples. In contrast, the accuracy ranged between 98.4 and 100.2% for day 1 and 98.6 and 103.2% for day 2. These results also complied with the ICH guidelines. The recovery of ALB from the powder complex was 97.59% ALB. The RSD was equal to 0.9–1% for all measurements.

### 2.2. Characterization of the Complex

The results of the EE of the prepared formulations are indicated in Table 1. The results show that the kneading method was superior to the other methods. Both βCD and HPβCD in ratios of 1:2 ALB to CD gave the highest EE. However, other methods gave values close to these values. When the ratio was increased from 1:1 to 1:2, the EE was raised. Still, a further increase in the ratio of CDs resulted in a decrease in the percent encapsulation of ALB, which means that a further increase in the ratio of CDs will not cause a further increase in the EE of ALB.

**Figure 2:**
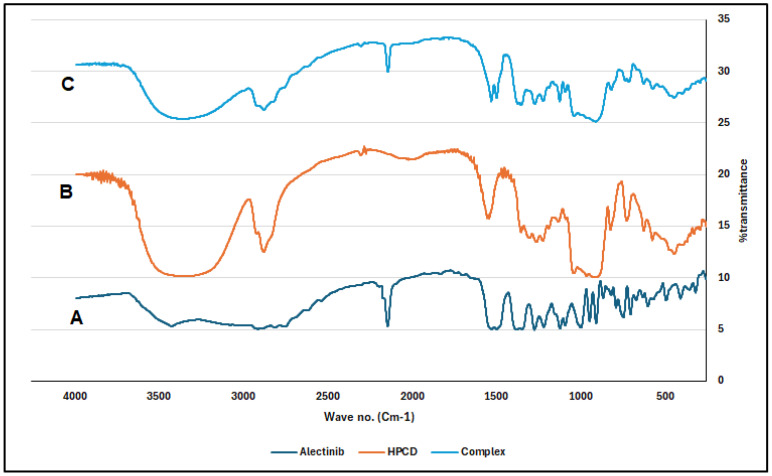
FTIR results: (A) ALB showing the major peaks of comparison (nitril bond stretching at 2220 cm^−1^; (B) HPβCD showing the 3395 cm^−1^ peak of O-H stretching; (C) the complex showing shortening of the nitril bond stretching and shortening and widening of the O-H stretching peak at the 3395 cm^−1^ as a result of interaction.

FTIR and DCS were used to identify the complex. According to the FTIR chart in Figure 2, the ALB-distinguishing peaks are as follows: 2220 cm^−1^ (nitrile bond stretching), 1490 cm^−1^ (N-H bending), 1600 cm^−1^ (C=C stretching), 1385 cm^−1^ (C-H stretching), 1660 cm^−1^ (C=O stretching), and 1250 cm^−1^ (C-N stretching). For HPβCD alone, the most frequent IR peaks are 3395 cm^−1^ (O-H), 2930 cm^−1^ (C-H stretching), 1628 cm^−1^ (H-O-H bending), 1156 cm^−1^ (C-O stretching), and 1032 cm^−1^ (C-O stretching and C-O-C stretching). 

**Figure 3:**
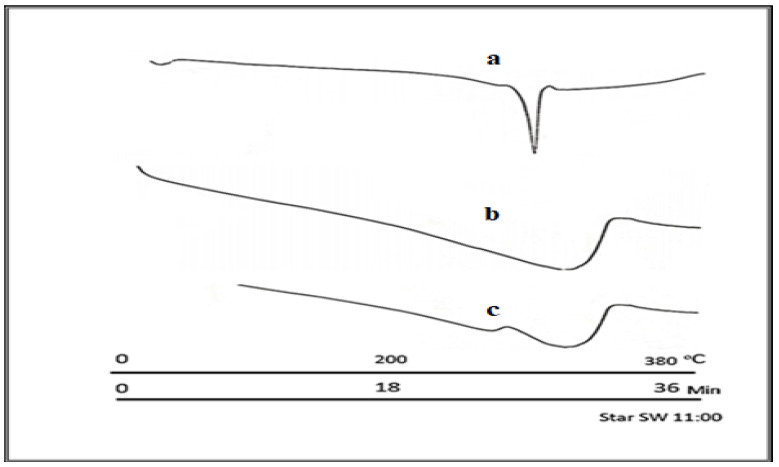
DSC thermogram: (a) ALB showing its melting point at 290 °C; (b) HPβCD with a wide, undefined peak in the range of 260–300 °C; (c) ALB–HPβCD complex with the disappearance of the ALB sharp peak inside the CD.

The characterization through DSC confirms the formation of the ALB–HPβCD (1:2) complex, as shown in Figure 3. ALB–HPβCD 1: The two complexes were selected as the formulation of the capsules to be studied. The DSC thermogram reveals the melting point of ALB to be around 290 °C. The complexes showed complete diminishing of the sharp melting point of ALB, and there were broad peaks at the range of 250–350 °C, which are explained as the physical interaction of ALB with either type of CD.

### 2.3. Phase Solubility Study

**Figure 4:**
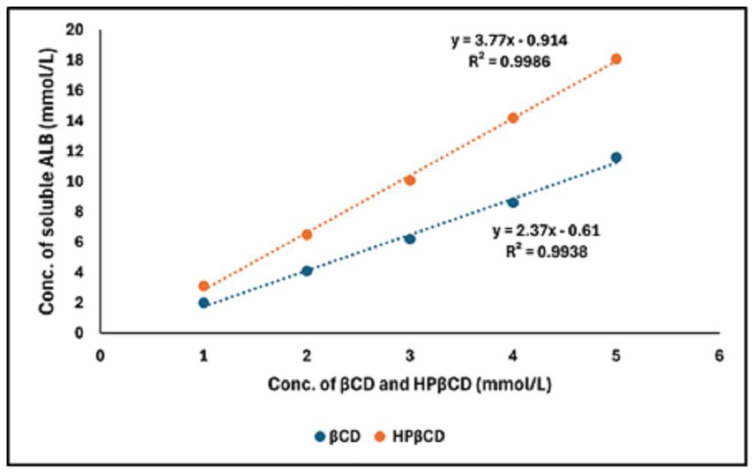
Phase solubility diagram of ALB with βCD and HPβCD. A solubility diagram of ALB with βCD; a solubility diagram of ALB with HPβCD and regression data.

Figure 4 shows the phase solubility profile of ALB–βCD. The solubility of pure ALB was estimated as 2.0 × 10^−3^ mmol (app. 1 µg/mL). The concentration in mmol/L of solubilized ALB was increased with an increase in the concentration of CDs linearly; this indicated a 1:1 stochiometric interaction of ALB with both types of CD. At the same concentrations, the ALB-in-HPβCD solution showed a relatively higher solubility than βCD. The Ks (stability constant) value was observed to rise from 500 M^−1^ for βCD to 1836 M^−1^ for HPβCD.

### 2.4. Computer Simulations of the Inclusion Complex

**Figure 5:**
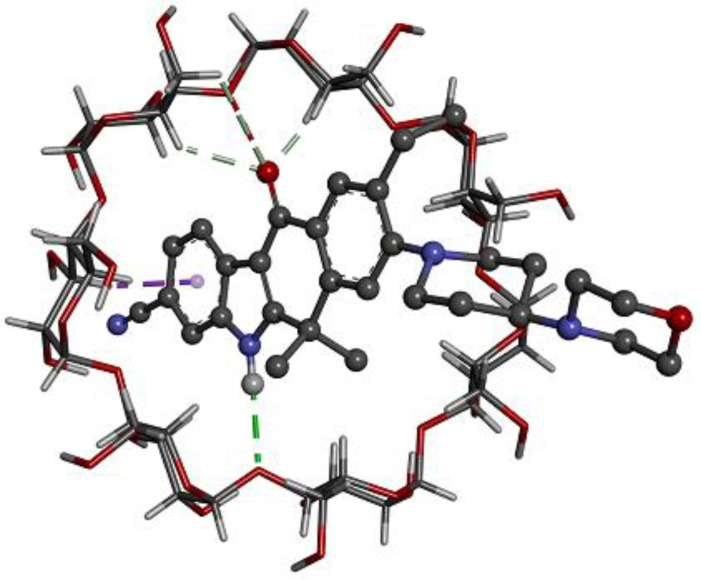
Interaction between ALB and βCD, as proposed by Gaussian09 software showing the possible sites of interaction between CD and ALB (ALB: black balls (C atoms), red balls (O atoms), blue balls (N atoms), grey balls (H atoms); CD, grey-red rods (C-O bond), grey-black rods (C-H bond)); green dotted line (interaction of piperidine hydrogen with CD oxygen); purple dotted line (hydrophobic interaction) and grey dotted line (interaction of CD hydrogen atoms with oxygen atom of the morpholine).

**Figure 6:**
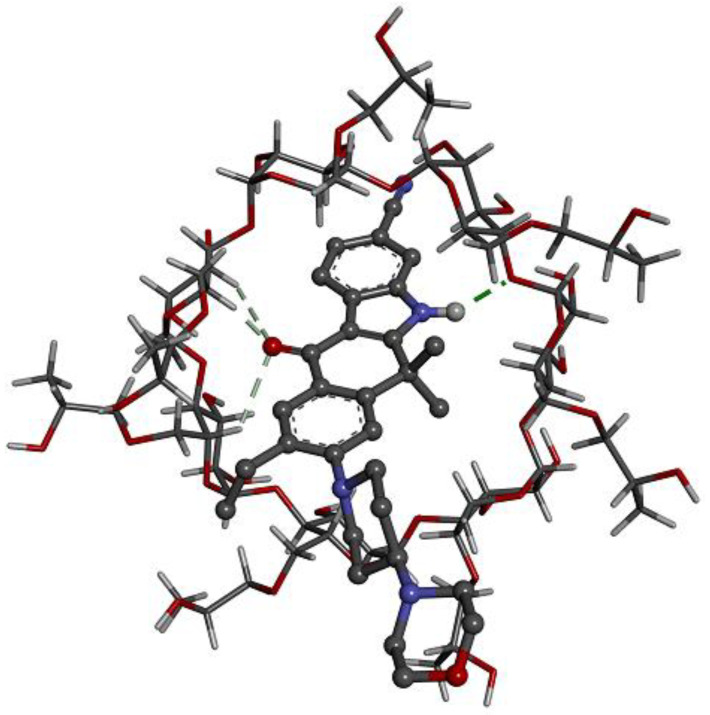
Interaction between ALB and HPβCD, as proposed by Gaussian09 software showing the possible sites of interaction between HPβCD and ALB (ALB: black balls (C atoms), red balls (O atoms), blue balls (N atoms), grey balls (H atoms); CD, grey-red rods (C-O bond), grey-black rods (C-H bond)); green dotted line (interaction of piperidine hydrogen with CD oxygen); purple dotted line (hydrophobic interaction) and grey dotted line (interaction of CD hydrogen atoms with oxygen atom of the morpholine).

Docking calculations produce a quantitative value of the binding affinity of different complexes and provide valuable insights into the conformation of the guest–host complexes. ALB exhibited the highest binding affinity (−7.29 kcal/mol) when complexing with HPβCD, followed by βCD (−6.21 kcal/mol), where it matched well with the experimental part obtained. Figure 5 and Figure 6 may provide a better understanding of the binding relationship between the host–guest complexes. The tetracyclic ring system with a fused indole ring is contained within the cone in both cases, while the morpholine moiety is located on the outer rim of the cyclodextrin cavity. The same results were obtained from the FTIR analysis.

### 2.5. Dissolution Study

**Figure 7:**
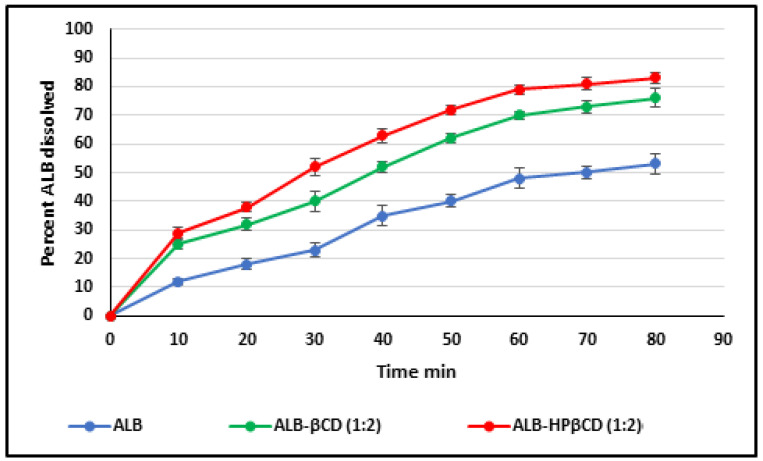
The dissolution of ALB powder, ALB–βCD (1:2) powder, and ALB–HPβCD (1:2) powder at 37 °C, 100 rpm, USP dissolution apparatus type II.

The dissolution rate profiles of standard ALB alone, ALB–βCD, and ALB–HPβCD (1:2) complex in pH 6.8 at 37.0 ± 0.5 °C are presented in Figure 7. The release rate profiles were expressed as the percent cumulative of ALB released from the complexes against time in min. The plot shows an improvement in the ALB’s dissolution rate released from ALB–βCD (1:2) powder and ALB–HPβCD (1:2) powder over ALB pure powder. This indicates that ALB’s solubility improved by forming complexes with βCD and HPβCD. Taking T30 (amount released in 30 min) as a point of comparison, the results showed a significant increase in dissolved ALB from the βCD (32%) and HPβCD (52%) complexes concerning pure ALB powder (18%) (*p* < 0.05) for both.

The application of different release models to the dissolution of the powder gave the results shown in Table 2. Zero-order, first-order, Peppas, Higuchi, and Hixson–Crowell models were all fitted on the results. The results showed that free ALB release followed a zero-order pattern, probably because of ALB’s low solubility. The release from the complex showed the highest correlation with the first-order model. This is due to an increase in ALB’s solubility and dissolution rate by the effect of the complexation process.

### 2.6. In Vivo Bioavailability Study

#### 2.6.1. Method of Analysis and Validation

**Figure 8:**
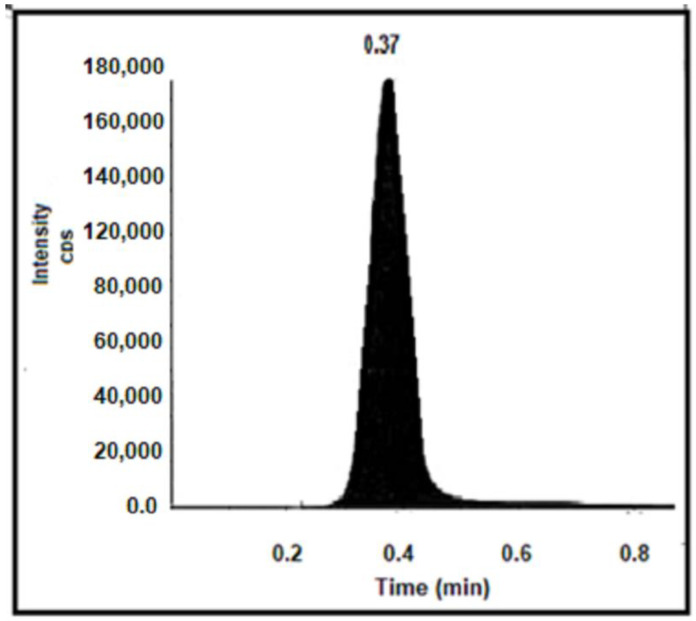
Extracted ion chromatogram of ALB in rat plasma (mass 483.3/396.3 Da).

The retention time (RT) of ALB in rat plasma was 0.37 min, as shown in Figure 8, which makes the method quick and time-saving.

The within-run precision and accuracy were determined by analyzing six samples with three replicates on the same day. The concentration and precision were within the approved %RSD limits of less than 15. Additionally, the accuracy required by established criteria, namely 85–115% for all concentrations, was met according to the ICH. The data accuracy suggested that ALB in plasma may be quantified appropriately across all concentration levels. The lower limit of quantification (LLOQ) results demonstrate that the analytical technique for ALB can accurately determine concentrations within the LLOQ. The accuracy and precision of ALB in rat plasma are shown in Table 3.

R^2^ was equal to 0.9993 for linearity, and the back calculation gave a %RSD between 1.2 and 3.8 for the LLOQ. In addition, the accuracy was also calculated to be 95.8–106.2%. The range was from 15.0 to 4000 ng/mL.

The recovery of ALB from various spiked samples was calculated using the QC Low, QC Mid, and QC High concentrations in addition to the LLOQ. The results showed that the recovery of ALB ranged from 92.7% to 98.5%, with an average of 96.8%, and the RSD was equal to 2.74, which is accepted by the ICH guidelines.

#### 2.6.2. Bioavailability Parameters

**Figure 9:**
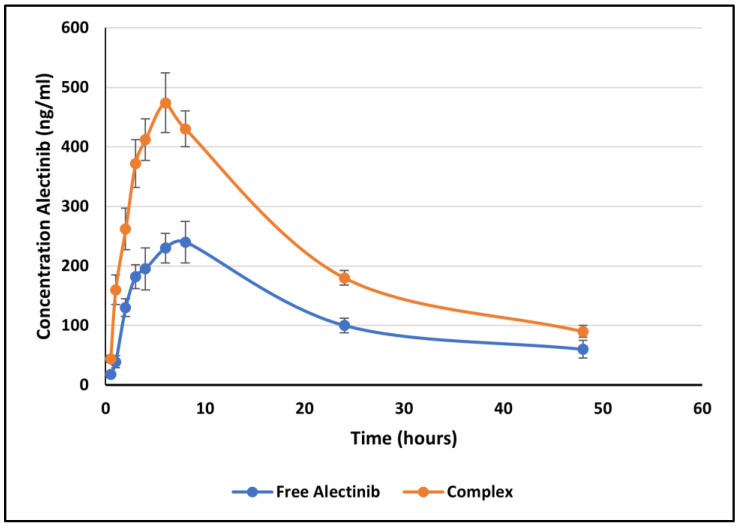
Plasma level–time profile of ALB from F1 (ALB without complexation) in G1 and from F2 (ALB in complex form with HPβCD) in G2 showing the difference in Cmax (474 and 240 ng/mL) and Tmax (5.1 and 7.33 h), which are significant for both the 0.05 and 0.1 CI levels.

The plasma level–time profile of F1 and F2 is depicted in Figure 9, and Table 4 illustrates the bioavailability parameters (Cmax, Tmax, and AUC0-48) and the elimination rate constant of ALB, as determined by NCA. The concentrations of ALB at 72 h were below the LLOQ.

Tmax is used in bioavailability and drug absorption studies to determine the medication absorption rate. AUC is used to determine the extent of drug absorption, whereas Cmax is used to determine both the rate and extent. ALB was prepared as an HPβCD inclusion complex in this investigation to promote dissolution and solubility, hence enhancing bioavailability. The in vitro dissolution results indicated an increase in dissolution, corresponding to the rise in drug bioavailability.

## 3. Discussion

The results showed that the kneading method was much better than other methods in the encapsulation of ALB with βCD and HPβCD. Optimal encapsulation efficiency was achieved at a 1:2 ALB-to-CD ratio, presenting stoichiometry’s importance for efficient drug encapsulation. However, going beyond this ratio produced diminishing returns, which implied that a careful balance is needed for efficient formulation. The FTIR shortening of the 2220 cm^−1^ peaks of ALB, which corresponds to the ALB nitrile group, indicates that the nitrile group might be included inside HPβCD. The short, wide peak of 3398 cm^−1^ in the complex compared to the original peak of HPβCD indicated that the cyclodextrin’s O-H group may have formed a connection with ALB. In general, the alterations in the IR spectra depicted in Figure 3 might indicate some kind of physical interaction of ALB with HPβCD. The absence of new peaks of new chemical entities proved the absence of chemical interactions. For HPβCD, DSC revealed a broad peak from 280 to 340 °C. In the complex thermogram, the sharp peak of ALB is embedded in HPβCD and is not well visible, indicating the inclusion of complex formation between ALB and HPβCD. ALB formed a stable complex with both βCD and HPβCD, with the highest solubility with HPβCD in a ratio of 1:2. The phase solubility study showed a significant increase in ALB’s solubility with a 1:1 stochiometric ratio and a stability constant of 1836 M^−1^. These values indicate the formation of a relatively more solid inclusion complex of ALB–HPβCD than ALB–βCD. The stability constant (Ks) value was reported to be between 50 and 5000 M^−1^, considered the most favorable for enhancing the solubility and stability of poorly soluble drugs [28]. Finally, HPβCD improved the solubility of ALB significantly with good stability of the complex. ALB demonstrated better binding affinity with HPβCD (−7.29 kcal/mol) than βCD (−6.21 kcal/mol), as revealed by docking calculations, in line with the experimental results. Figure 6 and Figure 7 represent a visual illustration of the host–guest relationship and demonstrate the intracavity location of the tetracyclic ring system of ALB within the cyclodextrin cavities, consistent with the FTIR analysis results.

The dissolution results showed that ALB–βCD (1:2) powder and ALB–HPβCD (1:2) powder improved the dissolution rate of the free powder. Figure 9 shows that T30 increased from 18% of free ALB to 32% for ALB–βCD and 52% for ALB–HPβCD. Both types of complexes gave significantly higher percent release (*p* < 0.05) than free ALB. Comparing ALB–HPβCD (1:2) with ALB–βCD (1:2) powder, the ALB–HPβCD powder gave significantly higher percent drug release at 30 min (T30, *p* < 0.05) as well as total drug release at the end of the test. 

The Cmax of ALB in F1 was equal to 240 ± 26.95 ng/mL and increased to 474 ± 50.97 ng/mL when taken in F2, which contains the ALB–HPβCD complex. The AUC0-48 was equal to 5946.75 ± 265 for F1 and was increased to 10,520 ± 310 ng.h/mL for F2. The statistical analysis results indicated a significant rise in the AUC or amount of drug absorbed, roughly to double, which means that there was a higher amount of ALB absorbed from F2. Figure 9 further illustrates the differences in the parameters of F1 and F2. Tmax, which measures the rate of drug absorption, was shortened from 7.33 h to 5 h, meaning a shorter absorption time and improved rate. The Cmax was increased from 240 ± 26.95 ng/mL to 474 ± 50.97 ng/mL due to the increased extent and ALB absorption rate. The statistical differences were evaluated on 0.05 and 0.1 CIs, and on both levels, the increase in AUC and Cmax and the decrease in Tmax are all significant. Compared to pure ALB, the solubility of ALB in gastric and intestinal fluids (water) was significantly increased due to complex formation with HPβCD. Additionally, the solubility experiments revealed that the complex dissipated more rapidly than pure ALB. As a result, an increase in ALB bioavailability in complex form was anticipated. As a result of these data, the improvement of the bioavailability of ALB through this procedure might decrease the dose administered to patients, thus reducing the side effects of this drug.

The formation of inclusion complexes between ALB and βCD or HPβCD is a novel aspect of the current research. To the best of our knowledge, these specific inclusion complexes have not been previously reported. Previous studies have reported other approaches to enhance the solubility and dissolution of ALB. For example, Eun Ji Park et al. increased the solubility and dissolution of ALB using suspended self-nanoemulsifying drug delivery systems [29]. Sumit Kumar Saha et al. utilized surface modification dispersion techniques to enhance the solubility and dissolution of ALB [30]. However, the current study is the first to investigate inclusion complexes of ALB with βCD and HPβCD with the aim of enhancing solubility, dissolution rate, and oral bioavailability.

## 4. Methodology

### 4.1. Materials and Chemicals

Alectinib HCl (Selleck, Houston, TX, USA), methanol, DMSO (dimethyl sulfoxide) (HPLC grade, Merck, Rahway, NJ, USA), sodium lauryl sulfate (SLS) (Sigma-Aldrich, St. Louis, MO, USA), and rosuvastatin (Sigma, St. Louis, MO, USA), were all used in this study. 

### 4.2. Development of the Method of Analysis Based on UV Absorbance

The UV spectrophotometer was selected as a method of analysis to detect and quantify ALB alone and ALB in the inclusion complexes. ALB is practically insoluble in water, with a log *p*-value of 5.2. This study used absolute methanol, ethanol, and DMSO to make an ALB solution. The solutions with the best solubilization were obtained in methanol (0.5 mg/mL). Dilutions of 50 and 100 μg/mL were prepared using methanol and scanned from 200 to 400 nm using a UV spectrophotometer (model U-2000, Hitachi, Tokyo, Japan), and methanol was used as blank and control. The λmax was obtained at 340 nm, and to guarantee its specificity, the method for measuring ALB in the samples was validated by scanning βCD and HPβCD at 200–400 nm. The approach was verified for its precision, accuracy, linearity, recovery, and robustness. The linearity test employed calibration values ranging from 15, 25, 30, 50, 60, 70, 80, and 90 μg/mL. The absorbance was measured thrice, and the average ± standard deviation (SD) was detected at 340 nm. The percentage of RSD was also determined.

For precision and accuracy, the method’s precision was calculated by analyzing 6 samples with 3 replicates on day 1 (intra-day) and day 2 (inter-day). The %RSD was calculated by evaluating the ratios of SD to the mean. The accuracy of this procedure was determined by comparing the actual values found in the samples with the practical amounts from the controls. The acceptable value of %RSD should be less than 2.0%, according to the International Council for Harmonization (ICH) guidelines for technical requirements for Pharmaceuticals for Human Use. The recovery of the method was measured for the powder blend of ALB and all excipients used in the formulation of the capsules. Also, 70% and 130% ALB formulas were prepared to examine recovery at higher and lower amounts of ALB.

Various conditions were adopted for reliability to measure a sample of 70 μg/mL ALB. Conditions like varying the wavelength ± 5 nm, sample test warming to 30 °C, and altering the instruments from other labs were used. The solvent was not changed because ALB has a lower solubility in different solvents, and precipitation occurred upon trying. Each sample was read in triplicate, and % accuracy and %RSD were calculated. The % accuracy and %RSD were measured for each sample, read in triplicate.

### 4.3. Preparation of the ALB–CD Complex

βCD and HβCD derivatives were used in this study. Three methods with four ratios wt./wt. (1:1, 1:2, 1:3, and 1:4) ALB:CDs were used for the preparation of the ALB–CD complex. The kneading method was performed using a mortar and pestle and 50 mg of ALB, and the specified weight of CD was mixed with a gradual addition of distilled water until a paste was formed. Mixing in one direction was continued for one hour. The samples were dried in an oven at 40 °C, collected, and placed in Eppendorf tubes for characterization and analysis. The solvent evaporation method dissolved ALB in methanol (50 mg/100 mL). The designated quantity of βCD or HPβCD was dissolved in 50 mL of distilled water. The prepared solutions were combined using a magnetic stirrer, and the resulting mixture was agitated for ten hours. A rotary evaporator at 40 °C removed the solvent with continuous stirring at a reduced pressure. After that, the final product was obtained by drying the sample in an oven (37 °C/48 h). The mixture was crushed and sieved (mesh sieve #65) and stored in a tightly closed container.

The spray drying method was also used to prepare the required complex (Buchi Nano Spray Dryer B-90 HP, Flawi, Switzerland). ALB was dissolved in 50 mL of methanol, following which HPβCD or βCD was dissolved in 50 mL of distilled water. The two mixtures were combined by sonication for 20 min to provide a clear, final solution. Later, the solution underwent a spray-dry process, and the drying conditions were as follows: temperature (outlet), 90 °C; temperature (inlet), 168 °C; flow rate (solution), 1000 mL/h; flow rate (air), 400 N1/h. The final inclusion complex was crushed and sieved using sieve mesh size #65 and kept in an air-tied container.

### 4.4. Characterization of the Prepared Complexes

The prepared complexes were characterized by complexation or encapsulation efficiency (EE), Fourier-Transform Infrared (FTIR), and Differential Scanning Calorimetry (DCS).

The EE (%) was calculated using the equation below (1):

EE(%) = (amount of ALB in the complex × 100)/(the total amount of ALB)(1)

The amount of encapsulated ALB was determined by dissolving a specific wt. of the dried complex in water and filtration to obtain the soluble complex. Then, methanol was added to dissolve the complexed ALB due to its higher affinity to it than water. The sample was put on the magnetic stirrer for one hour after the optimization of the process, using 200 rpm, and covered to protect it from light. The samples were analyzed using the validated method, and then their concentration was recorded three times with the SD. The FTIR analyses used the FTIR (SHIMADZU EUROPA-LC-2030C PLUS, Duisburg, Germany) instrument and the KBr disc method. Also, DSC used a DSC (Mettler Toledo 1 star system) instrument, and the temperature range was 4–400 °C at 10 °C/min. Both tests were applied to ALB, βCD, HPβCD, and the complex with the highest percent encapsulation efficiency (EE%). 

### 4.5. Phase Solubility Study

Phase solubility tests were conducted in distilled water following the procedure outlined in the Higuchi and Connors technique [31]. An excess amount of ALB was added into 20 mL of distilled water containing different amounts of βCD and HPβCD. Samples were placed in the water bath (25 °C/2 days/50 rpm) to attain equilibrium. Samples of 3 mL were withdrawn and filtered through a 0.45 μm nylon membrane filter. The filtrate (1 mL) was diluted appropriately with methanol, stirred for one hour (200 rpm), and assayed at 340 nm. The concentrations used were 2, 4, 10, 15, and 20 mmol/L for both βCD and HPβCD. The solubility of pure ALB was also examined by using distilled water and an excessive amount of ALB under identical conditions. After constructing a phase solubility graph, the apparent stability constants (*Kst*) were determined using the equation below:

(2) $$Kst=\frac{slope}{So\;(1-slope)}$$

where *So* is the absolute solubility of ALB in the absence of CD.

### 4.6. Computer Simulations of the Inclusion Complexes

The structure of the guest molecule, ALB, was downloaded as a mol file from the ChemSpider database (www.chemspider.com, accessed on 6 January 2022). The structure was optimized using the AM1 method (Gaussian09 software, version 4.2.6) [32]. In contrast, the 3D structure of the host molecule, βCD, can be retrieved from the Cambridge Crystallographic Data Center (CCDC). The structure of the lipid membrane was verified, and the crystallized molecules were separated. Moreover, hydroxypropyl groups were grafted by using GaussView (The Molinspiration Database) to complete the hydroxypropyl-βCD structure. Then, the structure of the guest molecule was optimized; the optimized structure was docked to the center of the host molecules, βCD and HPβCD. For docking calculations, the Kollman United Atom (KUA) charge parameters were added to the complexes using Autogrid as part of Autodock 4 software in a grid box of 60 × 60 × 60 dimensions (Molinspiration Database). The list of all possible conformations of the guest molecule was docked onto the center of the conformation of the host molecule by using the Lamarckian genetic algorithm, with 250 runs for each guest molecule. 

### 4.7. Dissolution Test

In the following circumstances, the dissolution test was carried out on the prepared ALB capsules and the complex-containing ones. The dissolution medium was a USP type II (paddle type) and 900 mL of pH 6.8 phosphate buffer at 37 ± 1 °C with the rotation speed fixed at 100 h. At selected time points (10, 20, …, 80), 2 mL samples of dissolution medium were withdrawn and replaced with a fresh buffer. Times start from 0-time points for powder dissolution and end after 20 min (average time to dissolve the capsule shell).

### 4.8. In Vivo Study

#### 4.8.1. LC–MS/MS for the Detection of ALB in Rat Plasma

A calibration procedure was developed and validated for LC–MS/MS analysis of the ALB in the serum of rat samples. The Shimadzu SCL-10A VP system control (Kyoto, Japan) has a mass detector (LCMS2010A) from Shimadzu Kylimatu, Japan. The mobile phase comprised acetonitrile–deionized water–formic acid (70: 30: 0.1%, *v*/*v*). Rosuvastatin was utilized as an internal standard. The injection volume was 10 µL; the flow rate was 400 µL; the autosampler temperature was 5 °C; the column temperature was 25 °C; and the column was ACE (C18; 4.6 × 50 mL) with particle size of 5.0 µm.

#### 4.8.2. Extraction Method 

The samples were prepared for injection into the instrument as follows: Samples (including known/unknown spiked plasma and samples) were obtained from the deep freezer, followed by a period of room-temperature thaw. Later, a plasma pipet was performed on the samples after they were shaken to form a mixture. A total of 270 µL of blank plasma per 300 µL of spiked plasma were pipetted into the labeled test tubes, and 30 µL of serial solutions into blank plasma were added. Then, 50 µL of rosuvastatin (IS) working solutions were added. Then, the samples were vortexed for roughly 10 s. A total of 700 µL of acetonitrile (precipitation solutions) were added and vortexed again for 2.0 min, then centrifugated at 4000 rpm for roughly five min at room temperature. After that, 200 µL from the supernatant was transferred to the HPLC vials inserts. Finally, the vials were capped and transferred into the autosampler racks.

#### 4.8.3. Method Validation 

The following tests were executed to validate the method according to the ICH guidelines: recovery, accuracy, precision, linearity, and the detection limit [33]. 

The concentrations of ALB used in the linearity test ranged between 15 and 4000 ng/mL, and they were 15, 500, 1000, 2000, 3200, and 4000 ng/mL. Readings were made in triplicate, and each sample’s average ± SD was calculated. The AUC ratio ALB/IS was then tested for linear regression by calculation of the correlation coefficient (R2), and %RSD was then evaluated according to the acceptance criteria of the ICH guidelines. The accuracy and within-run precision were determined by examining six samples with three replicates. The RSD values were determined as a percentage of the standard deviation to the average ratios. The actual accuracy of the procedure was measured and given by plotting the practically obtained values from the control samples against the theoretical value reading inside the sample. The concentrations used were the QCLow (130 ng/mL), QCMid (2200 ng/mL), and QCHigh (3000 ng/mL). The LLOQ for ALB was established at 15 ng/mL. The LLOQ was established based on a theoretical estimate of the lowest concentration the drug may reach before being eliminated from the body at the time of the experiment. As a result, the technique can be relatively accurate for measuring lower concentrations in test samples. The coefficient of variation (CV) percent of back-calculated calibration standard concentrations was also calculated. 

#### 4.8.4. Percent Recovery 

ALB can be described as the part of the analyte amount added or presented to the analytical part of the test substance that is extracted and represented for measurement purposes (ICH, 2022). Three samples at low, medium, and high QC concentrations were extracted and then analyzed. Also, three extracted blank samples spiked with the analyte at low, medium, and high concentrations were analyzed using the developed method.

The recovery of ALB in samples was calculated as follows:

(3) $$Recovery\;\%=\frac{Area\;of\;extracted\;plasma\;sample}{Area\;of\;blanks\;spiked\;with\;the\;analyte\;post-extraction}\times 100$$

The CV percent of analyte recovery in the (low, medium, or high) range must be less than 20%.

The selectivity was detected by assessing only plasma (the blank sample) at time zero before giving the drug.

### 4.9. Pharmacokinetic Study

The study protocol was validated and approved by the Institutional Review Board (IRB) of Applied Science University (ASU) (Decision no. 6/2021–2022). The preclinical investigation was conducted at the Animal House of ASU as a combined project. A total of 12 Wistar rats were prepared for this study and divided into two groups, with each group containing 6 rats. The average weight of the animals was 200 g ± 15 g, aged four weeks, and they were all males. Group 1 (G1) received 15 mg/Kg of free ALB, and group 2 (G2) received the equivalent of 15 mg/kg of the complex for each animal. 

The animals for the experiment were prepared by depriving them of food (for about twelve hours) apart from full access to water, and they were identified by a sign mark on the tail. After the dosing of ALB, samples were retrieved from the rat’s tail. After, the tail was warmed for half a minute via a hot pad before every sampling period. A total of 350 µL every time was withdrawn on the following time intervals: 0, 0.5, 1, 2, 3, 4, 6, 8, 24, and 48 h. The samples were kept in heparinized Eppendorf tubes, centrifugated to obtain the plasma, frozen at −25 °C, and kept until the time of analysis.

### 4.10. Non-Compartmental Analysis of Plasma Concentration–Time Data 

The plasma level–time profile of ALB was obtained using Microsoft Excel. Two profiles, F1 in G1 and F2 in G2, were plotted. Data were treated by Phoenix WinNonlin (version 8.3) to obtain the basic bioavailability parameters (Cmax, Tmax, and AUC0-t) in addition to Kel and the half-life of ALB. ANOVA was applied to detect significant parameter differences using a 10% confidence interval.

## 5. Conclusions

This study used a complexation concept with βCD and HPβCD to increase the solubility and dissolution rate of ALB, a tyrosine kinase inhibitor important in non-small-cell carcinoma treatment. The inclusion complexes, confirmed through FTIR and DSC analyses, demonstrated stability, with the most significant solubility improvement observed at a 1:2 (ALB:HPβCD) ratio. Computer simulations validated this complexation’s efficacy, especially in improving in vitro dissolution. Studies in Wistar rats yielded a significant increase in bioavailability without changing the elimination rate constant. The interaction of ALB with HPβCD becomes an effective strategy. It seems to be a promising approach to improving oral administration and developing the curative potency of ALB in treating non-small-cell carcinoma.

## Figures and Tables

**Table 1:** The EE of the prepared formulations.

Type of Complex	Ratio of ALB:CD	Percent EE (%)
Kneading Method		
ALB–βCD	1:1	42.5 ± 2.0
ALB–βCD	1:2	64.0 ± 2.5
ALB–βCD	1:3	63.5 ± 1.0
ALB–βCD	1:4	58.6 ± 1.6
ALB–HP βCD	1:1	45.6 ± 1.3
ALB–HP βCD	1:2	65.2 ± 1.5
ALB–HP βCD	1:3	62.5 ± 0.8
ALB–HP βCD	1:4	56.8 ± 1.0
Solvent evaporation method		
ALB–βCD	1:1	38.5 ± 0.5
ALB–βCD	1:2	54.6 ± 2.6
ALB–βCD	1:3	50.5 ± 1.0
ALB–βCD	1:4	35.4 ± 3.0
ALB–HP βCD	1:1	35.0 ± 1.2
ALB–HP βCD	1:2	49.0 ± 2.6
ALB–HP βCD	1:3	45.3 ± 1.5
ALB–HP βCD	1:4	42.6 ± 2.1
Spray Drying		
ALB–βCD	1:1	58.3 ± 2.3
ALB–βCD	1:2	61.2 ± 0.6
ALB–βCD	1:3	60.5 ± 0.5
ALB–βCD	1:4	55.3 ± 2.4
ALB–HP βCD	1:1	55.6 ± 0.5
ALB–HP βCD	1:2	63.5 ± 1.5
ALB–HP βCD	1:3	55.1 ± 2.7
ALB–HP βCD	1:4	49.2 ± 1.0

**Table 2:** Correlation of the fitness of dissolution data on release models.

Model	Zero Order	First Order	Peppas Model	Higuchi Model	Hixson–Crowell Model
Free ALB					
R^2^	0.985	0.961	0.977	0.980	0.972
ALB released from the complex (ALB–HPβCD 1:2)					
R^2^	0.931	0.989	0.972	0.970	0.900

**Table 3:** Results of in-day precision and accuracy.

Samples (*n* = 6)	LLOQ 15.0 ng/mL	QC_Low_ 130.0 ng/mL	QC_Mid_ 2200.0 ng/mL	QC_High_ 3000.0 ng/mL
Mean	14.70	131.63	2183.01	2967.55
SD	0.39	4.48	65.47	24.41
CV%	2.6	3.6	2.9	0.8
Accuracy	98.2%	101.3%	99.2%	98.9%

**Table 4:** Bioavailability parameters and elimination rate constants of F1 (formula without complex) and F2 (formula with complex).

Formula	C_max_ (ng/mL)	T_max_ (h)	AUC-48 (ng. h/mL)	AUC-Inf (ng. h/mL)	Kel (h^−1^)
F1 (free alectinib)	240 ± 26.95	7.33 ± 1.03	5946.75 ± 265	7280.02 ± 306	0.045 ± 0.008
F2 (complex)	474 ± 50.97	5.1 ± 1.3	10520 ± 310	12320 ± 415	0.050 ± 0.002

## Data Availability

Data are contained within the article.
